# The impact of culturally-informed messages to reduce sugar-sweetened beverage consumption: An experiment among Black women in the United States

**DOI:** 10.1371/journal.pone.0312361

**Published:** 2024-11-26

**Authors:** Rhyan N. Vereen, Marissa G. Hall, Francesca Dillman Carpentier, Rachel W. Goode, Seth M. Noar, Allison J. Lazard

**Affiliations:** 1 Hussman School of Journalism and Media, University of North Carolina at Chapel Hill, Chapel Hill, North Carolina, United States of America; 2 Gillings School of Public Health, University of North Carolina at Chapel Hill, Chapel Hill, North Carolina, United States of America; 3 Lineberger Comprehensive Cancer Center, University of North Carolina at Chapel Hill, Chapel Hill, North Carolina, United States of America; 4 Carolina Population Center, University of North Carolina at Chapel Hill, Chapel Hill, North Carolina, United States of America; 5 School of Social Work, University of North Carolina at Chapel Hill, Chapel Hill, North Carolina, United States of America; University of Minnesota School of Dentistry, UNITED STATES OF AMERICA

## Abstract

**Objective:**

Sugar-sweetened beverage (i.e., sugary drink) consumption is associated with chronic health issues that disproportionately affect Black women. Culturally-informed (CI) health campaigns may be more effective among Black women than campaigns designed for general audiences. This study assesses the effects of a CI campaign on consumption intentions, comparing these effects to general audience and control campaigns.

**Methods:**

We conducted an online between-persons randomized experiment with a national convenience sample of 502 Black women in February 2023. Participants were randomly assigned to view a CI, general audience, or control campaign. Outcomes were intentions to decrease sugary drink consumption (primary outcome; range 1–7), knowledge of (range 0–4) and perceived susceptibility to health harms (range 1–5) and sharing intentions (range 0–3).

**Results:**

The CI campaign had significantly higher perceived cultural relevance (M = 4.61) than the general audience (M = 3.64) or control (M = 3.66; p’s<0.05) campaigns. Consumption intentions did not differ by campaign condition (F = 0.03, p>0.05). There was no main effect of campaign condition on knowledge or perceived susceptibility (p’s>0.05), though findings were moderated by body size. Based on body size, women reported less perceived susceptibility or knowledge when exposed to the CI campaign, compared to either the control or general audience campaign. Sharing intentions did not differ by campaign condition.

**Conclusions:**

Future research should continue to examine the role of refining message content over a longer duration to understand whether the anticipated impact of CI messages can be achieved in the context of sugary drink consumption among Black women.

## Introduction

Consuming sugar-sweetened beverages (i.e., sugary drinks) increases one’s risk of chronic conditions like weight gain, type II diabetes, and some cancers, that can lead to premature morbidity and mortality [1]. Consumption in the United States has been decreasing since the early 2000’s [2, 3], but daily intake of sugary drinks remains higher among Black women [4, 5], who have two times higher odds of consuming sugary drinks than white women [6]. Disproportionate consumption habits play a role in disparities in health outcomes [7], making sugary drink consumption a public health concern.

Health campaigns are one tool to address such concerns. Campaigns, alone, are unable to address disparities due to structural inequities, like more frequent marketing of sugary drinks and other unhealthy foods to Black neighborhoods [8, 9]. However, findings from several evaluations suggest that sugary drink consumption-related outcomes can be altered through campaign interventions. For example, evaluations of individual campaigns among adults saw increases in intentions to reduce sugary drink consumption [10, 11], trends toward or observed decreases in sugary drink consumption [12, 13], and decreases in sugary drink sales [14, 15]. According to a scoping review, sugary drink reduction social marketing and health promotion campaigns were positively associated with short-term outcomes (e.g., increased beliefs, knowledge, and intention) and behavioral outcomes (e.g., reduced sugary drink consumption and/or increased water consumption) [16]. Other reviews echoed these findings, concluding that the effect of campaigns among adults is small [17], but present [18].

However, limitations in these campaigns ability to address disparities in sugary drink consumption among Black women exist. Existing sugary drink reduction campaigns have been designed for large scale dissemination in a whole county or state [12, 14, 19] or samples where the racial makeup was relatively homogenous or unclear [15, 20]. Even when adequate numbers of Black women are included in study samples, it is uncommon for study findings to be disaggregated by both sex and gender, making it difficult to determine interventions’ effects among Black women [12, 19]. Therefore, the effect of sugary drink reduction messages developed for and among Black women remains unknown.

Culturally-informed messages could be especially well-equipped to address disparities [21–23]. Culturally-informed messages account for the shared ideas, beliefs, and experiences of a specific group, like Black women. However, few studies have tested culturally informed messages to reduce sugary drink consumption specifically among Black women.

Thus, the purpose of this study was to examine the impact of different campaign content on intentions to consume fewer sugary drinks, as well as secondary outcomes theoretically known to predict behavior change (i.e., perceived susceptibility, knowledge, and message sharing intentions) [18, 24–26].

## Methods

### Campaign design

**Fig 1:**
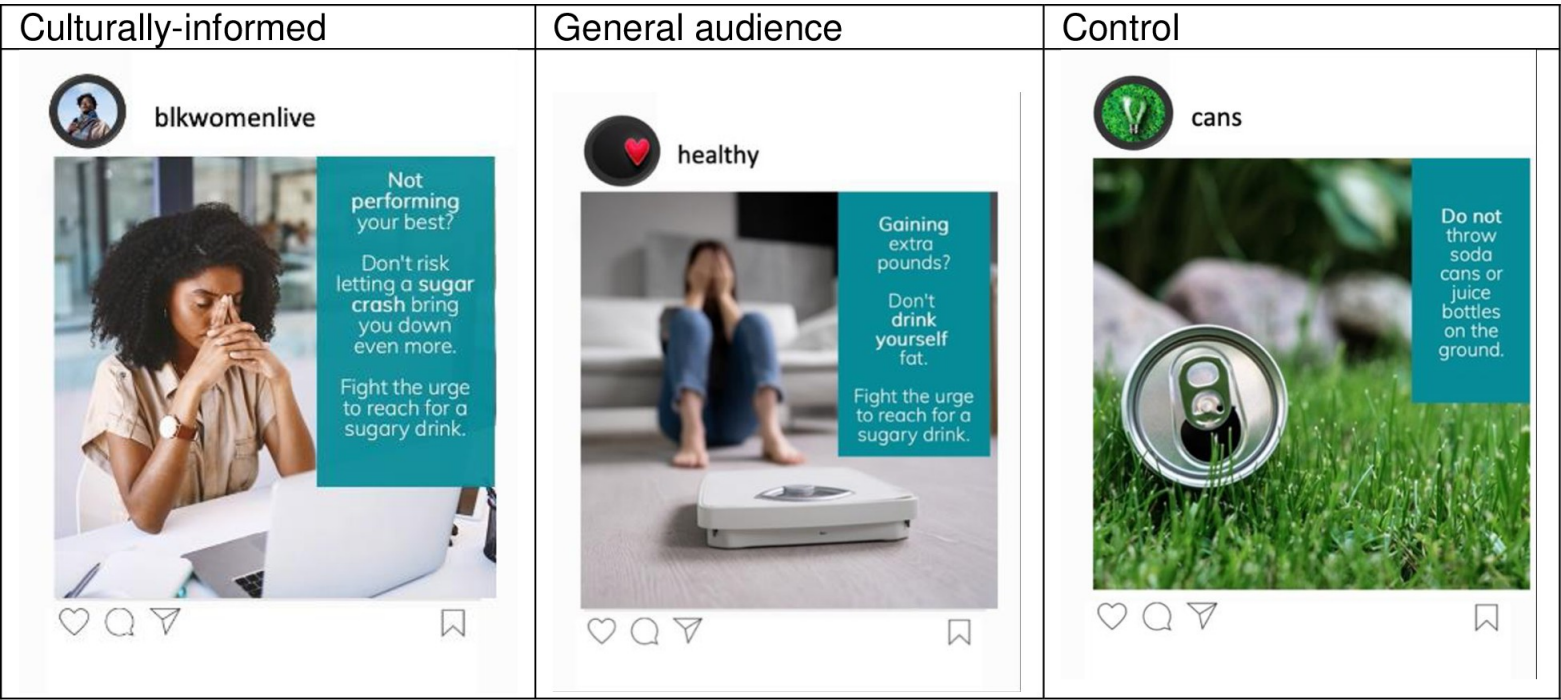
Sample of study stimuli by content condition.

We conducted a between-persons experiment with three experimental campaign content conditions: culturally-informed, general audience, and a control. Each campaign was comprised of 3 messages, each with text and an image. Each message was designed to mimic an Instagram post, like those posted on the Centers for Disease Control and Prevention (CDC)’s Instagram page [27]. A sample of campaign stimuli is presented in Fig 1.

Robust formative research was conducted to advise the development of the culturally-informed campaign. We used a formative literature review to identify previously established culturally-informed weight management intervention components, eating behaviors, and health and body-related perceptions among Black women. The review yielded four topical themes: *Family*, *Appearance*, *Mental well-being*, and *Spirituality*. Messages were drafted, then discussed and refined in a community feedback session. Perceptions of the refined messages were assessed in an online survey of Black women. Promising messages were used for the culturally-informed campaign. A more detailed report of formative research is presented in supplementary materials (S1 File). The general audience content condition used text from or similar to that of existing sugary drink campaigns, which focused on informational weight and/or health-based content [28, 29]. Text for the control was limited to messages about recycling or littering.

Campaign images were selected to correspond with the text in each individual message. All images were selected from online stock image databases. All images in the culturally-informed campaign contained someone one who appeared to be a Black woman. An effort was made to include Black women with various skin shades, hair styles, and in different settings. Images in the general audience campaign contained a woman of a different assumed race. Both campaigns have one image with a woman smiling, one image with a woman frowning, and one image with a woman covering her face. Control images were of soda cans and/or bottles on the ground.

### Inclusion criteria

Participants were recruited using CloudResearch Prime Panels in February 2023. Prime Panels include an aggregate of panels, comprised of individuals who have opted-in to complete market research surveys. Prime Panels has previously been used in social science research and is known to implement in-house data checks to ensure data quality [30, 31]. Panel members who identified as African American, female, and living in the United States were sent a link to the screener to determine full eligibility for participation in this study.

Eligible participants were 18 years or older, self-identified as female, Black or African American, and reported consumption of sugary drinks. Consumption was defined as consuming at least one serving per week of one or more sugary drinks. Adapted items from the BEVQ-15 beverage frequency questionnaire were used [32]. The scale asks participants to indicate how often they consume a list of 7 sugary drinks- soda, fruit drinks, sports drinks, energy drinks, sweetened ready-to-drink (RTD) teas, sweetened RTD coffee, and flavored water [33]. Individuals who reported consumption of at least one of the drinks at least once per week were eligible. Given an interest in consumption behaviors among young and middle-aged adults, where consumption is higher [4, 34], individuals over 65 years old were not eligible. The screener was accessed 710 times. A total of 565 individuals were eligible, 548 individuals consented, and 520 participants completed the survey.

### Procedure

The experiment was conducted via an online, mobile-friendly questionnaire programmed in Qualtrics software. Participants provided consent by reading the consent form online, agreeing that they had read the form, and selecting the icon to move forward to the online questionnaire. They were then randomly assigned by the Qualtrics randomizer function to one of the three experimental conditions. Participants viewed one message at a time from their respective condition in a random order. After each message, participants were asked questions about perceived cultural relevance and sharing intentions. After viewing all three messages, they were asked about intention to consume fewer beverages, knowledge, and perceived susceptibility, followed by items about health, body size, and demographics. Participants were provided an incentive by CloudResarch at the completion of the questionnaire. This study was deemed exempt by the University of North Carolina at Chapel Hill Institutional Review Board.

### Measures

#### Perceived cultural relevance

As an experimental manipulation check, the survey assessed perceived cultural relevance with the following statements after each message, “This message was made to appeal to people like me”, “This message was made to appeal to Black women”, and “This message is culturally relevant for Black women”. Responses were provided on a 7-point scale ranging from disagree completely (1) to agree completely (7). The mean of the three items was used for analyses (α = 0.92).

#### Intention

To assess intentions to consume fewer sugary drinks, participants responded to the statements, “In the next week, I [want to/ plan to/ am likely to] drink fewer sugary drinks” [11]. Responses were provided on a 7-point scale from not at all likely (1) to very likely (7). The mean of the three items was used for analyses (α = 0.90).

#### Perceived susceptibility

To measure perceived susceptibility of health harms, participants were asked what the chances of them getting these four health concerns (i.e., obesity, diabetes, tooth decay and heart disease) “as a result of drinking sugary drinks”. Response options ranged from no chance (1) to very good chance (5). The mean of the items was used for analyses (α = 0.89).

#### Knowledge

To measure knowledge of health harms, participants were asked whether sugary drink consumption contributes to the following four health concerns: obesity, diabetes, tooth decay and heart disease [35]. Response options were true, false, or don’t know. Responses were coded as correct (true; 1) or not (false or don’t know; 0) and then correct responses were summed for a range between 0 and 4.

#### Sharing intentions

Participants were asked whether they would share the message with a friend, sibling, spouse, parent, other family member, other person, or an exclusive response of no one. Responses were dichotomized as no one (0) versus anyone (1). The number of messages participants were willing to share with someone was summed for a range between 0 and 3.

#### Body size

Body mass index (BMI), used to screen for health risks, was calculated using self-reported height and weight. BMI was dichotomized as overweight or obesity (BMI at or above 25) versus not having overweight or obesity (BMI<25; hereafter, “healthy weigh”).

### Statistical analyses

All analyses were conducted in SAS 9.4. Then, we validated whether participants perceived the culturally-informed campaign as more culturally relevant than the other campaigns (p < .05). Descriptive characteristics of participants were reported using frequencies and percentages for categorical variables and means and standard deviation for continuous variables.

We conducted a one-way analysis of variance (ANOVA) to determine whether the outcomes (intention to consume, knowledge, perceived susceptibility, sharing intention) differed by condition. If significant (p>0.05), Tukey pairwise tests were conducted to identify statistically significant differences in means between conditions.

Previous research identified differential findings in the effect of sugary drink reduction campaigns and related outcomes by weight categories [13] and concerns about unintended effects of health messages on weight stigma [36, 37]. Therefore, we performed exploratory moderation analyses to determine the robustness of findings among different weight categories (overweight or obesity versus not). We sought to determine whether the effect of campaign condition, particularly the culturally-informed condition, on all four outcomes differed by body size category. Proc GLM was used to assess this interaction in an unbalanced ANOVA. A significant interaction (p<0.05) indicated evidence that the effect of the condition on the outcome was dependent on body size. A non-significant interaction (p>0.05) indicated that the effect of the condition on the outcome was not dependent on body size. We visually probed the nature of the interaction by plotting the means by experimental condition for significant interactions.

## Results

Participants (N = 520) were, on average, 37.4 (SD = 13.2) years old (Table 1). All participants identified as female and Black or African American, with some participants also identifying as Hispanic or Latina (4.6%), American Indian or Alaska Native (1.0%), or Middle Eastern or North African (0.6%). Participants lived in one of 39 states, in urban areas (48.8%), with many financially living comfortably (29.3%) or getting by (38.3%). Most perceived themselves as having excellent, very good, or good health (67.3%) and had a calculated BMI categorized as overweight or having obesity (68.2%).

**Table 1:** Participant characteristics, N = 502.

Characteristics		N or Mean	% or SD
Age		37.4	13.2
Gender			
	Female	165	100.0
Race and ethnicity*			
	Black or African American	165	100.0
	Hispanic or Latina	23	4.6
	American Indian or Alaska Native	5	1.0
	Middle Eastern or North African	3	0.6
Economic stress			
	Living comfortably	147	29.3
	Getting by	192	38.3
	Finding it difficult	109	21.7
	Finding it very difficult	54	10.8
Rurality			
	Urban	245	48.8
	Suburban	183	36.5
	Rural	74	14.7
Sugary drink consumption			
	One time per week	288	57.4
	More than one time per week	214	42.6
Perceived health status			
	Poor or fair	164	32.7
	Excellent, very good, or good	338	67.3
BMI, missing = 27			
	Not overweight or obese	151	31.8
	Overweight or obese	324	68.2

*Percentages may not total 100%, as participants were instructed to select all that apply.

### Impact of campaign condition

The culturally-informed campaign had significantly higher perceived cultural relevance (M = 4.61, SD = 1.65) than the general audience campaign (M = 3.64, SD = 1.77; p<0.05) and the control (M = 3.66, SD = 1.63; p<0.05) content conditions.

Intention to consume fewer sugar drinks did not differ by content condition, with similar means in the culturally-informed (M = 4.86), general audience (M = 4.87), and control (M = 4.83) conditions; F = 0.03, p = 0.975; Table 2). Similarly, neither perceived susceptibility nor knowledge differed by content condition (F = 0.54, p = 0.586 and F = 0.30, p = 0.743, respectively). On average, participants were willing to share more messages from the culturally-informed (M = 2.37) and control (M = 2.33) campaigns than the general audience campaign (M = 2.12), though not statistically significant (F = 2.44, p>0.05).

**Table 2:** Effects of message content condition on outcomes.

	Intention	Perceived susceptibility	Knowledge	Sharing intentions
Content condition	Mean (SD)	Mean (SD)	Mean (SD)	Mean (SD)
Culturally-informed	4.86 (1.7)	3.33 (1.2)	2.71 (1.4)	2.37 (1.0)
General audience	4.87 (1.8)	3.42 (1.3)	2.80 (1.3)	2.12 (1.2)
Control	4.83 (1.6)	3.46 (1.3)	2.81 (1.3)	2.33 (1.1)
F	0.03	0.54	0.30	2.44
p-value	0.975	0.586	0.743	0.088

Intention ranges from 1 to 7; Perceived susceptibility ranges from 1 to 5; Knowledge ranges from 0 to 4; Sharing intentions ranges from 0 to 3.

### Moderation by body size

**Fig 2:**
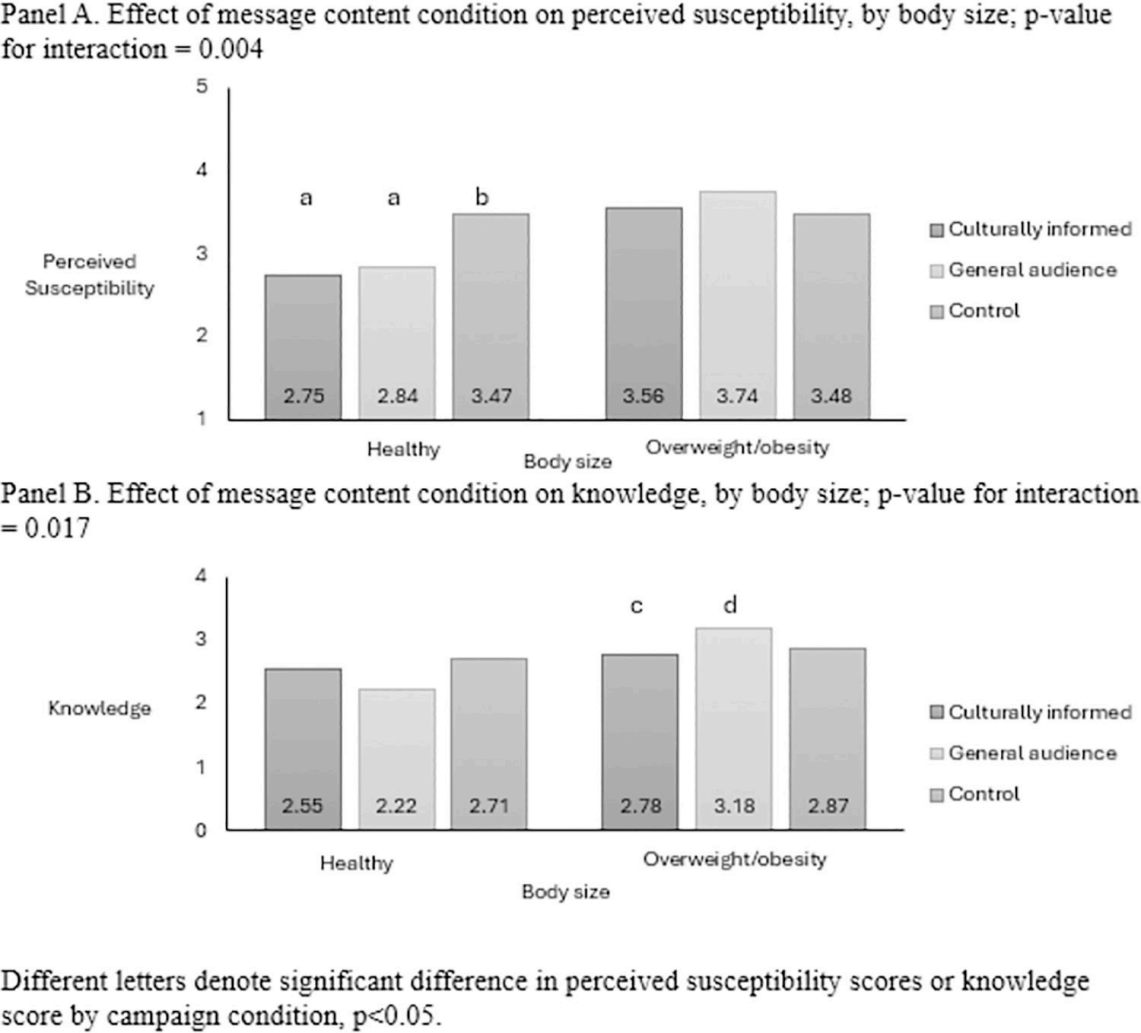
Effect of message content condition on perceived susceptibility and knowledge by body size.

Body size moderated the impact of perceived susceptibility (p for interaction = .0.004) and knowledge (p for interaction = 0.017; Fig 2), but not intentions to consumer fewer sugar drinks (p for interaction = 0.451) or sharing intentions (p for interaction = 0.061). Among women with healthy weight, perceived susceptibility to health harms was higher in the control arm (M = 3.47) compared to the culturally-informed (M = 2.75, p<0.05) or general audience arms (M = 2.84, p<0.05); there were no differences by condition among women with overweight/obesity (Fig 2A). Among women with overweight/obesity, knowledge scores were higher in the general audience campaign condition (M = 3.18) than the culturally-informed campaign (M = 2.78, p<0.05; Fig 2B).

## Discussion

This study examined the impact of message campaign condition on intention to consume fewer sugar drinks, as well as perceived susceptibility and knowledge of health harms and sharing intentions. There were no significant differences in intentions to consume fewer sugary drinks between culturally-informed, general audience, and control conditions. Moderation analyses detected differences in the effect of campaign condition on perceived susceptibility and knowledge by body size. Sharing intentions did not differ by experimental condition.

Other studies have shown that general audience messages can reduce in sugary drink consumption outcomes [10–18], but this study did not. There are many possible explanations for this. First, this is the first study, to my knowledge, to focus solely on and therefore, have a full sample of Black women. Therefore, we were able to determine the campaign effect among the prioritized population, Black women, while the overall effect reported in previous campaigns was representative of the samples with aggregated racial and gender groups. Our findings could also be due to the campaigns themselves. For example, its possible the culturally-informed campaign lacked deep level tailoring and was not different enough from the other campaigns [38] and/or that there needed to be a different level of campaign exposure to see a difference in campaign effects [39]. Finally, social determinants of health like disproportionate advertising for sugary drinks in Black neighborhoods and access to sugary drinks pose additional barriers to effective obesity reduction efforts among Black women [40] that a campaign alone cannot compensate for. A campaign, in combination with evidence-based multi-level public health efforts like warning labels [41, 42], sugar tax [43, 44], and other measures that decrease access [45] are likely needed. Research should continue to assess the individual effect of campaigns, in addition to the combined effects of these interventions among Black women.

Secondary outcomes, perceived susceptibility and knowledge, did not differ by content condition but were moderated by body size. Risk perceptions, which differ by perceived body size [46], may explain some of the differences. But the differential effects by campaign by body size are interesting. Specifically, among women with healthy weight, women who saw the culturally-informed or general audience campaigns had lower scores of perceived susceptibility of health harms than those who saw the control. This finding was unexpected and concerning, given that in an ideal situation, the culturally-informed and general audience campaigns would have increased, not lowered, perceived susceptibility scores. One explanation is that these women may have embodied a present-orientation, which happens to be more common among Black adults [23, 47, 48] combined with optimism bias, and believed that since they currently consumed sugary drinks and were a healthy weight and not experiencing the health harms in question (e.g. obesity, heart disease) that they must not be susceptible. However, this belief is a concern because it can negatively affect behavior and has the potential to exacerbate harms among a group that is already experiencing disparities.

Among women categorized as overweight/obesity, knowledge scores were lower for those who viewed the culturally-informed campaign than those who viewed the general audience campaign. This finding was less worrisome, as it was likely due to the concordance in the health harms inquired about in the knowledge measure and the health harms mentioned in the general audience campaign. However, it important to acknowledge because it suggests that the campaigns perhaps worked as expected among women who had overweight/obesity, but had similar outcome among those with healthy weight. Perhaps women with overweight/obesity were previously exposed to messages about health harms related to sugary drink consumption (e.g., those in the general audience campaign) or found the culturally-informed campaign less relevant. The mechanisms behind these moderated findings are beyond the scope of the current study, but should be assessed and explored in future research.

In general, participants were willing to share campaign messages. This could be a positive finding because campaign generated interpersonal communication is generally associated with positive change [49], including plans to reduce sugary drink consumption [18]. However, this finding did not differ by campaign. Questions remain about how to increase message sharing, if not by campaign content, and the context around sharing, including the perceived sentiment of the message (positive or negative) and intentions to persuade or counter persuade.

Study limitations must be considered. This was a cross-sectional study with controlled and brief exposure to sugary drink campaign content. Therefore, we were unable to discern how study outcomes may have (or have not) been different if participants had been exposed to content in a natural digital platform setting over a longer period of time. There were also potential limitations with the campaigns developed for this study. Given the presence of null findings, it is possible that content of the campaigns were too similar. Messages in the culturally-informed campaign had higher perceived cultural relevance, but a deeper level of tailoring in the culturally-informed campaign messages may be needed for messages to be effective. Messages also included images of fair-skinned Black women with natural hair. This use of this racially ambiguous image of Black women is growing in use in advertising, despite the fact that it perpetuates stigma against darker skinned women and those with African features [50]. We may have also designed a control that was too strong by using images of soda cans and bottles that could have cued beliefs about sugary drinks similarly to the other treatment condition stimuli. Despite these limitations, we recruited a large national sample (N = 502) of Black women with varying body sizes (i.e., had obesity, overweight, or normal weight) and contrary to many studies that claim to evaluate the effect of culturally relevant interventions, we intentionally collected data to support differences in perceived cultural relevance across study conditions.

## Conclusion

We filled a gap in the literature by developing a culturally-informed campaign for sugary drink reduction and determining the effect of this campaign, in addition to general audience and control campaigns, among Black women. In this study, neither the culturally-informed campaign nor a general audience campaign performed better than a control at influencing intentions to consume fewer sugary drinks. Furthermore, some women, based on their self-reported body size, reported less perceived susceptibility or knowledge—known antecedents to behavior change—when exposed to the culturally-informed campaign, compared to either the general audience or control campaign. Findings bring about questions about the message needs for Black women with various body sizes and the relationship between cultural relevance and behavior change. As new campaigns are developed, the inclusion of Black women should be ensured, and findings disaggregated to grow knowledge about the effects of sugary drink campaigns for Black women and other populations experiencing disparities. Future research should also continue to examine the role of refining message content over a longer duration to understand whether the anticipated impact of culturally-informed messages can be achieved in the context of sugary drinks.

## Supporting information

S1 File Identification of culturally-informed themes. (DOCX)
