# Supplementary material for: The impact of culturally-informed messages to reduce sugar-sweetened beverage consumption: An experiment among Black women in the United States
Source: PLoS One. 2024 Nov 26;19(11):e0312361. doi: 10.1371/journal.pone.0312361 (PMC11594584; doi:10.1371/journal.pone.0312361)
Supplement: S1 File — (DOCX) [file pone.0312361.s001.docx]

**Supplemental Materials:** “The Impact of Culturally-informed Messages to Reduce Sugar-Sweetened Beverage Consumption: An Experiment among Black Women”

**Identification of Culturally-Informed Themes**

We used a formative literature review to identify previously established culturally relevant weight management intervention components, eating behaviors, and health and body-related perceptions among Black women. The review yielded four topical themes: *Family, Appearance, Mental well-being*, and *Spirituality*. Themes and rationale are presented in Figure 1.

Figure 1. Culturally-Informed Themes and Rationale

| **Theme** | **Rationale** |
| --- | --- |
| Family | Black women are known to prioritize the wellbeing of those around them above themselves (1). In Black communities, this could look like preparing a homecooked meal for one’s spouse, which is a considered symbol of love (2) and is a predictor of marital satisfaction and stability (3). It could also look like taking on the heavy feeling of responsibility for what their children consume (4). Given that eating behaviors typically begin in the home with loved ones (5) and Black women acknowledge themselves as role models,(4) empowering them to initiate positive change in consumption habits in the home may be beneficial. |
| Appearance | Health campaigns tend to have an underlying focus on weight loss and/or being thin. However, many Black women desire to have a thick and curvy physique (6–8). Other physical features could, instead, be highlighted in health campaigns. For example, Black women are sensitive to perceptions of their hair, skin, and facial features (9–11). A focus on the direct and or indirect effects of sugar on features that uniquely affect Black women, like hair loss (via disproportionate burden of diabetes)(12,13), increased risk of hyperpigmentation from facial acne than white counterparts (14,15), or dental caries (via disproportionate presence of tooth decay)(16) should be considered. |
| Mental well-being | Whether it is acting out the “superwoman schema”(1,17) or falling into the expectations of “strong Black woman”(18), Black women are frequently expected and encouraged to disregard negative experiences and emotions and push through. This tends to negatively affect the mental well-being of these women. The depression and stress that may be caused are positively associated with emotional eating among Black women. (19,20) Instead of ignoring hardships, messages could both acknowledge difficult circumstances and empower women to stay true to themselves as they attempt to consume fewer sugary drinks. |
| Spirituality | Spirituality is a belief that the purpose of life is greater than oneself. Spirituality among Black women dates back to slavery, when it was used to transcend traumatic experiences and invoke hope (21,22), It continues to be used to protect against negative feelings and emotions among Black women (23) and is positively associated with quality of life (24,25). Spirituality can, but does not always, include religion. Similar to other religiosity-tailored messages (26,27), messages may point to God’s creation of nature and preventive health tools or how spirituality can be coupled with these tools to maintain a healthy lifestyle. |

**Message Selection**

This study was approved by the University of North Carolina at Chapel Hill Institutional Review Board.

We drafted 5 messages per theme, for a total of 20 messages. Messages were comprised of two parts, a culturally-informed statement characterized by text referencing the message’s respective theme and a call to action (e.g., put down the sugary drinks). Messages were pretested during a community feedback session with participants who identified as Black, female, and consumed at least one sugary drink per week (N=6). Using feedback from the group, three out of the five messages in each theme were selected for further evaluation in a survey. See the final messages from the survey in Table 1.

Next, we conducted an online survey. All participants (N=165) identified as female and Black or African American (Table 1). Few (4%) also identified with another race or ethnicity. The average age was 32 years old (SD=4.83).

An adapted version of the UNC Perceived Message Effectiveness (PME) scale was used as the primary outcome for the study.(28) PME is often used to identify messages that have the potential to change behavior (28,29); this measure is generally considered to be sensitive enough to detect minor differences between similar messages, yet predictive of longer-term behavior change (30). The UNC PME scale has been used extensively for nutrition messaging research (31–34). Scores range from 1 (not at all) to 5 (a great deal). PME was highest for the Appearance theme (Mean=3.12), specifically the “tooth fairy” (M=3.26) and “acne” (M=3.24) messages.

Cultural relevance scores range from 1 (completely disagree) to 7 (completely agree). Higher scores represent higher perceptions of cultural relevance. Sharing intentions scores range from 1 (Very unlikely to share) to 5 (Very likely to share). Themes and individual messages averaged just above the middle of the cultural relevance (range 1-7) and sharing intentions (range 1-5) scales, with cultural relevance themes averaging between 4.03 to 4.29 and messages averaging between 3.91 to 4.60 and sharing intentions themes averaging between 3.36 to 3.45 and messages averaging between 3.27 to 3.60.

Messages with the highest PME were used to develop a culturally-informed campaign to be used in an experiment.

Table 1. Mean scores for perceived message effectiveness (PME), cultural relevance, and sharing intentions by theme and message

| **Theme** |  | **PME** | **Cultural relevance** | **Sharing intentions** |
| --- | --- | --- | --- | --- |
| **Brief name** | **Message** | **Mean (SD)** | **Mean (SD)** | **Mean (SD)** |
| Family | | 2.90 (1.10) | 4.09 (1.55) | 3.45 (1.03) |
| *What you give* | Your family will drink what you give them. Try to keep sugary drinks out of your home. | 3.04 (1.24) | 4.09 (1.72) | 3.60 (1.26) |
| *Be an example* | Be an example for your family. Try to consume fewer sugary drinks. | 2.96 (1.20) | 4.05 (1.67) | 3.49 (1.22) |
| *My boo* | The only sugar you need in this house is from me, boo. Let’s keep sugary drinks out of our home. | 2.72 (1.30) | 4.12 (1.87) | 3.28 (1.38) |
| Appearance | | 3.12 (1.07) | 4.29 (1.52) | 3.44 (1.05) |
| *Tooth fairy* | Getting a visit from the tooth fairy as an adult is not as exciting as getting a visit as a child. Too much sugar can damage your teeth. Put the sugary drinks down. | 3.26 (1.24) | 4.16 (1.70) | 3.56 (1.29) |
| *Acne* | Help get rid of acne by drinking fewer sugary drinks. | 3.24 (1.25) | 4.14 (1.68) | 3.52 (1.27) |
| *Sugar scrubs* | Sugar scrubs, not sugary drinks, keep my Black beautiful. Leave those sugary drinks at the store. | 2.88 (1.28) | 4.60 (1.83) | 3.27 (1.32) |
| Mental well-being | | 3.00 (1.12) | 4.04 (1.57) | 3.36 (1.06) |
| *Sugar crash* | Not feeling your best? Don’t let the sugar crash bring you down even more. Put down the sugary drinks. | 3.11 (1.22) | 4.00 (1.67) | 3.35 (1.32) |
| *Mind set* | A new mind set requires a new habit. Try drinking something other than the sugary drinks. | 3.00 (1.25) | 4.18 (1.64) | 3.44 (1.29) |
| *Sweet life* | You don’t need sugar to make life sweet. Put down the sugary drink and go enjoy life. | 2.92 (1.28) | 3.91 (1.79) | 3.28 (1.26) |
| Spiritual | | 2.94 (1.06) | 4.03 (1.47) | 3.40 (1.00) |
| *Replenish* | Replenish your body. Replenish your soul. Reduce your consumption of sugary drinks. | 2.99 (1.19) | 4.15 (1.62) | 3.48 (1.24) |
| *Natural form* | Try to consume things in their natural form. Let sugary drinks go. | 2.87 (1.15) | 3.91 (1.64) | 3.31 (1.34) |
| *Sugar high* | The sugar high is nowhere near as wonderful as your higher calling. Be the best version of yourself. Leave the sugary drinks behind. | 3.02 (1.28) | 4.07 (1.72) | 3.42 (1.22) |

Perceived message effectiveness (PME) scores range from 1 (not at all) to 5 (a great deal). Cultural relevance scores range from 1 (completely disagree) to 7 (completely agree). Higher scores represent higher perceptions of cultural relevance. Sharing intentions scores range from 1 (Very unlikely to share) to 5 (Very likely to share).

References

1. Woods-Giscombé CL. Superwoman Schema: African American Women’s Views on Stress, Strength, and Health. Qual Health Res. 2010 May;20(5):668–83.

2. James DCS. Factors influencing food choices, dietary intake, and nutrition-related attitudes among African Americans: application of a culturally sensitive model. Ethn Health. 2004 Nov;9(4):349–67.

3. Haynes FE. Gender and family ideals: An exploratory study of Black middle-class Americans. Journal of Family Issues. 2000;21:811–37.

4. Thompson D, Callender C, Velazquez D, Adera M, Dave JM, Olvera N, et al. Perspectives of Black/African American and Hispanic Parents and Children Living in Under-Resourced Communities Regarding Factors That Influence Food Choices and Decisions: A Qualitative Investigation. Children (Basel). 2021 Mar 18;8(3):236.

5. Gillespie AH, Gillespie, Jr. G. Family Food Decision-making: An Ecological Systems Framework. Journal of Family and Consumer Sciences. 2007;99(2).

6. Cameron NO, Muldrow AF, Stefani W. The Weight of Things: Understanding African American Women’s Perceptions of Health, Body Image, and Attractiveness. Qual Health Res. 2018 Jul 1;28(8):1242–54.

7. Smith I, White BM. Barriers and Facilitators to Adhering to the American Heart Association’s Life’s Simple 7 for African American Women Living in Public Housing. Journal of Health Care for the Poor and Underserved. 2021;32(4):2012–29.

8. Peterson M. Nutrition Practices and Obesity Standards Among Obese, African American Women [Internet]. [United States -- Minnesota]: Walden University; 2021 [cited 2022 Aug 5]. Available from: https://www.proquest.com/docview/2544898941/abstract/D53DF2DC7C374CCBPQ/1

9. Capodilupo CM, Kim S. Gender and race matter: The importance of considering intersections in Black women’s body image. Journal of Counseling Psychology. 2014;61:37–49.

10. Awad GH, Norwood C, Taylor DS, Martinez M, McClain S, Jones B, et al. Beauty and Body Image Concerns Among African American College Women. Journal of Black Psychology. 2015 Dec 1;41(6):540–64.

11. Watson LB, Lewis JA, Moody AT. A sociocultural examination of body image among Black women. Body Image. 2019 Dec 1;31:280–7.

12. Sadler GR, Meyer MW, Ko CM, Butcher C, Lee S, Neal T, et al. Black Cosmetologists Promote Diabetes Awareness and Screening Among African American Women. Diabetes Educ. 2004 Jul 1;30(4):676–85.

13. Coogan PF, Bethea TN, Cozier YC, Bertrand KA, Palmer JR, Rosenberg L, et al. Association of type 2 diabetes with central-scalp hair loss in a large cohort study of African American women. International Journal of Women’s Dermatology. 2019 Sep 1;5(4):261–6.

14. Romańska-Gocka K, Woźniak M, Kaczmarek-Skamira E, Zegarska B. The possible role of diet in the pathogenesis of adult female acne. Postepy Dermatol Alergol. 2016 Dec;33(6):416–20.

15. Penso L, Touvier M, Deschasaux M, Szabo de edelenyi F, Hercberg S, Ezzedine K, et al. Association Between Adult Acne and Dietary Behaviors: Findings From the NutriNet-Santé Prospective Cohort Study. JAMA Dermatology. 2020 Aug 1;156(8):854–62.

16. CDC. Oral Health Surveillance Report: Trends in Dental Caries and Sealants, Tooth Retention, and Edentulism. Atlanta, GA: Centers for Disease Control and Prevention, US Department of Health and Human Services; 2019.

17. Knighton JS, Dogan J, Hargons C, Stevens-Watkins D. Superwoman Schema: a context for understanding psychological distress among middle-class African American women who perceive racial microaggressions. Ethnicity & Health. 2022 May 19;27(4):946–62.

18. Watson NN, Hunter CD. “I Had To Be Strong”: Tensions in the Strong Black Woman Schema. Journal of Black Psychology. 2016 Oct 1;42(5):424–52.

19. Pickett S, McCoy TP. Effect of Psychosocial Factors on Eating Behaviors and BMI Among African American Women. Clin Nurs Res. 2018 Nov 1;27(8):917–35.

20. Pickett S, Burchenal CA, Haber L, Batten K, Phillips E. Understanding and effectively addressing disparities in obesity: A systematic review of the psychological determinants of emotional eating behaviours among Black women. Obesity Reviews. 2020;21(6):e13010.

21. Lewis LM. Spiritual Assessment in African-Americans: A Review of Measures of Spirituality Used in Health Research. J Relig Health. 2008 Dec 1;47(4):458–75.

22. Musgrave CF, Allen CE, Allen GJ. Spirituality and Health for Women of Color. Am J Public Health. 2002 Apr;92(4):557–60.

23. Baxter K, Medlock MM, Griffith EEH. Hope, Resilience, and African-American Spirituality. In: Medlock MM, Shtasel D, Trinh NHT, Williams DR, editors. Racism and Psychiatry: Contemporary Issues and Interventions [Internet]. Cham: Springer International Publishing; 2019 [cited 2022 Sep 20]. p. 141–56. (Current Clinical Psychiatry). Available from: https://doi.org/10.1007/978-3-319-90197-8_8

24. Levin JS, Chatters LM, Taylor RJ. Religious effects on health status and life satisfaction among black Americans. J Gerontol B Psychol Sci Soc Sci. 1995 May;50(3):S154-163.

25. Borges CC, dos Santos PR, Alves PM, Borges RCM, Lucchetti G, Barbosa MA, et al. Association between spirituality/religiousness and quality of life among healthy adults: a systematic review. Health and Quality of Life Outcomes. 2021 Oct 21;19(1):246.

26. Kreuter MW, Lukwago SN, Bucholtz RDDC, Clark EM, Sanders-Thompson V. Achieving cultural appropriateness in health promotion programs: targeted and tailored approaches. Health Educ Behav. 2003 Apr;30(2):133–46.

27. Haughton MWK Lorna T. Integrating Culture Into Health Information for African American Women - Matthew W. Kreuter, Lorna T. Haughton, 2006. American Behavioral Scientist [Internet]. 2016 Jul 27 [cited 2021 Oct 22]; Available from: http://journals.sagepub.com/doi/abs/10.1177/0002764205283801

28. Baig SA, Noar SM, Gottfredson NC, Boynton MH, Ribisl KM, Brewer NT. UNC Perceived Message Effectiveness: Validation of a Brief Scale. Ann Behav Med. 2018 Oct 15;53(8):732–42.

29. Noar SM, Barker J, Bell T, Yzer M. Does Perceived Message Effectiveness Predict the Actual Effectiveness of Tobacco Education Messages? A Systematic Review and Meta-Analysis. Health Communication. 2020 Jan 28;35(2):148–57.

30. Baig SA, Noar SM, Gottfredson NC, Lazard AJ, Ribisl KM, Brewer NT. Incremental criterion validity of message perceptions and effects perceptions in the context of anti-smoking messages. J Behav Med. 2021 Feb 1;44(1):74–83.

31. Grummon AH, Hall MG, Taillie LS, Brewer NT. How should sugar-sweetened beverage health warnings be designed? A randomized experiment. Preventive Medicine. 2019 Apr 1;121:158–66.

32. Hall MG, Lazard AJ, Grummon AH, Higgins ICA, Bercholz M, Richter APC, et al. Designing warnings for sugary drinks: A randomized experiment with Latino parents and non-Latino parents. Prev Med. 2021 Jul;148:106562.

33. Hall MG, Grummon AH, Lazard AJ, Maynard OM, Taillie LS. Reactions to graphic and text health warnings for cigarettes, sugar-sweetened beverages, and alcohol: An online randomized experiment of US adults. Prev Med. 2020 Aug;137:106120.

34. Sigala DM, Hall MG, Musicus AA, Roberto CA, Solar SE, Fan S, et al. Perceived effectiveness of added-sugar warning label designs for U.S. restaurant menus: An online randomized controlled trial. Prev Med. 2022 Jul;160:107090.
